# Renal Tubular Epithelial Cell–Derived hsa_circ_0008925 From Urine Is Related to Chronic Renal Fibrosis

**DOI:** 10.1111/jcmm.70335

**Published:** 2025-01-12

**Authors:** Yuanhui Shi, Yuye Chen, Zihao Xiao, Yajie Wang, Cong Fu, Yuhan Cao

**Affiliations:** ^1^ Department of Nephrology Yi Ji Shan Hospital Affiliated to Wan Nan Medical College Wuhu Anhui China; ^2^ Anesthesia Laboratory and Training Center of Wannan Medical College Wuhu Anhui China; ^3^ Anhui Province Key Laboratory of Non‐Coding RNA Basic and Clinical Transformation Wuhu China; ^4^ Department of Cardiology Yi Ji Shan Hospital Affiliated to Wan Nan Medical College Wuhu Anhui China

**Keywords:** chronic kidney disease, circRNAs, mechanism, renal fibrosis, SRSF6

## Abstract

Renal fibrosis (RF) is a crucial pathological factor in the progression of chronic kidney disease (CKD) to end‐stage renal failure, and accurate and noninvasive assays to monitor the progression of renal fibrosis are needed. Circular RNAs (circRNAs) are noncoding RNAs that can be used as diagnostic biomarkers and therapeutic targets for human diseases. In this study, we analysed the expression of hsa_circ_0008925 in human urinary renal tubular cells and investigated its role in renal fibrosis. Urinary samples were collected from CKD patients with varying degrees of renal fibrosis; renal tubular epithelial cells were isolated from the urinary samples using magnetic bead sorting. In patients with moderate–severe renal fibrosis, the expression of hsa_circ_0008925 in urinary renal tubular epithelial cells was elevated compared to that in patients with no renal fibrosis to mild renal fibrosis. Spearman correlation analysis indicated that the hsa_circ_0008925 expression was positively correlated with serum creatinine (Scr, rs = 0.424, *p* = 0.031). The expression of hsa_circ_0008925 was elevated in TGF‐β1‐treated HK‐2 cells in vitro. Silencing of hsa_circ_0008925 using siRNA inhibited TGF‐β1‐induced fibrosis in HK2 cells. RNA pull‐down and mass spectrometric analyses indicated that serine/arginine‐rich splicing factor 6 (SRSF6) is the downstream of hsa_circ_0008925. Silencing mmu_circ_0002215 and inhibiting SRSF6 alleviated renal fibrosis in a UUO model in vivo. Inhibiting hsa_circ_0008925/SRSF6 alleviated renal fibrosis in vitro and in vivo. These findings suggest that targeting the hsa_circ_0008925/SRSF6 pathway could hold promise as a potential therapeutic strategy for treating renal fibrosis.

## 1 Introduction

Chronic kidney disease (CKD) is a major global public health issue, and its prevalence is increasing worldwide. The estimated global prevalence of CKD is 13.4%, while in China, it is approximately 10.8% [1]. Renal fibrosis is a common pathological feature of CKD leading to end‐stage renal disease (ESRD). Renal fibrosis is a complex pathological process involving multiple inflammatory molecules and proteins. The detailed mechanism of renal fibrosis still needs to be elucidated.

Circular RNAs (circRNAs) exhibit greater stability than linear RNAs due to their closed loop structure, allowing them to persist in various tissues and body fluids [2]. CircRNAs expression varies notably in different disease states, with dysregulation closely linked to the pathogenesis of numerous diseases [3]. Investigations into circRNAs in renal diseases have revealed their potential roles in regulating pathological processes such as renal inflammation, apoptosis, and fibrosis [4]. In our previous research, we found that urinary circRNAs can be used as noninvasive biomarkers of renal fibrosis based on chip analysis [5, 6]. Furthermore, we found that the expression of hsa_circ_0008925 in TGF‐β1‐treated HK‐2 cells increases synchronously with that of hsa_circ_0008925 in urinary exosomes [6]. This finding suggested that the hsa_circ_0008925 in urinary exosomes originates from tubular epithelial cells and may be involved in the pathological process of renal fibrosis. CircRNAs have been demonstrated to be key factors in regulating renal fibrosis both in vitro and in vivo [7]. Our previous study also suggested that hsa_circ_0008925 participates in the progression of renal fibrosis. However, the mechanism by which hsa_circ_0008925 regulates renal fibrosis still requires further elucidation.

In this study, we investigated the correlation between hsa_circ_0008925 derived from renal tubular cells in the urine and renal fibrosis through in vivo and in vitro studies and investigated its mechanism of action in renal fibrosis.

## 2 Materials and Methods

### 2.1 Study Population

This study was conducted at the Department of Nephrology, Yi Ji Shan Hospital, Wannan Medical College. A total of 26 patients with CKD of different stages and pathological types underwent renal puncture biopsy. The selection criteria for CKD patients were as follows: (1) age between 18 and 80 years, (2) availability of complete clinical case data and (3) no prior treatment with nonsteroidal drugs and/or immunosuppressants before biopsy. The exclusion criteria for CKD patients were as follows: (1) history of acute decline in renal function or acute and chronic urinary tract infection, (2) patients with chronic liver disease, cardiovascular diseases, malignant tumours or long‐term chemotherapy and (3) the need for kidney transplantation. All recipients signed an informed consent form. The Ethics Committee of Yi Ji Shan Hospital of Wannan Medical College approved this study (Approval number: 2022LS No. 77).

### 2.2 Collection of Clinical and Pathological Data

Basic demographic and clinical biochemical data of the CKD patients, including age, sex, blood pressure, Scr, cystatin C, blood urea nitrogen (BUN), estimated glomerular filtration rate (eGFR) and 24‐h urine protein, were collected. Additionally, renal histopathological data were obtained. Paraffin‐wrapped renal tissues were used to prepare 4‐μm sections for Masson staining. Twenty nonoverlapping fields per section were randomly selected at 200× magnification and photographed. The tubulointerstitial fibrosis areas were graded based on the percentage of total tissue area: no fibrosis–mild fibrosis (fibrotic area ≤ 25%) and moderate–severe fibrosis (fibrotic area> 25%). Glomerulosclerosis scoring was performed by calculating the average ratio of the number of sclerotic glomeruli to the total number of glomeruli in different microscopic fields of view.

### 2.3 Magnetic Bead–Sorted Cells

Morning urinary samples (100 mL) were collected from the included CKD patients. The urinary sediment cells were separated by centrifugation at 4°C and 3000× *g* for 30 min. Following the magnetic bead sorting protocol, a 0.5% bovine serum albumin buffer (Biofroxx, Germany) was used to resuspend the single‐cell suspensions. PE‐conjugated anti‐human CD13 antibody (Miltenyi, Germany) was added and incubated for 15 min at 4°C in the absence of light to label the urinary sediment cells. Subsequently, PE‐sorted magnetic beads (Miltenyi, Germany) were added and incubated for another 15 min. The resuspended single‐cell suspension was then added to the LD column (Miltenyi, Germany) of the magnetic field in the MACS separator (Miltenyi, Germany), and immunomagnetic bead sorting was performed. CD13^+^ cells were collected by passing the plunger and rinsing the positively selected cells with magnetic labelling. Similarly, CD11b antibody and the corresponding anti‐CD11b antibody (Miltenyi, Germany) were added separately. CD13^+^CD11b^−^ cells were finally collected.

### 2.4 Cell Culture, siRNA Transfection and Treatments

HK‐2 cells purchased from Procell (Pricella, China) were cultured in complete HK‐2 cell‐specific medium (Dulbecco's modfied Eagle's medium +10% foetal bovine serum +1% 100 U/mL penicillin and 100 μg/mL streptomycin; Pricella, China) in a humidified incubator with 5% CO_2_ at 37°C. HK‐2 cells were transfected with 50 nM hsa_circ_0008925 siRNA or 50 nM negative control siRNA (RiboBio, China) using Lipofectamine 2000 (SignaGen, USA) according to the manufacturer's protocol. Moreover, 20 μM of indacterol (a chemical inhibitor of SRSF6; Selleck Chemicals, USA) was used to inhibit SRSF6. Then, the cells were cultured for 24 h. HK‐2 cells (1 × 10^7^) were then exposed to 15 ng/mL of recombinant TGF‐β1 protein (Sino Biological, China) for 48 h. Total RNA or protein was collected from the cells.

### 2.5 RNA Pull‐Down

Initially, the collected cells were fixed with 1% formaldehyde (Sigma, Germany) for 10 min. Following this, the supernatant was collected after centrifugation by adding the mixed lysate (Thermo Fisher Scientific, USA) and subsequently treating the cells with ultrasonication until they were fully lysed and clarified. The subsequent probes were diluted to 100 μM and mixed in equal volumes with the protein‐rich supernatant. Subsequently, 100 μL of streptavidin magnetic beads (Thermo Fisher Scientific, USA) were added to capture the target proteins, which were rotated and mixed thoroughly before being incubated for 4 h. Then, the magnetic beads and protein complex were then separated via magnetic adsorption (Beyotime, China), the supernatant was discarded, and the beads were washed to eliminate unbound proteins. This washing step was repeated five times to ensure high purification. Next, 100 μL of loading buffer was added and incubated with shaking in a metal bath at 100°C for 10 min. Finally, the supernatant was centrifuged and subjected to mass spectrometry for the identification of proteins bound to the probe. Proteins that bind to hsa_circ_0008925 were detected using RNA pull‐down analysis performed by Hangzhou Hibio, China. The binding of proteins was determined by western blotting.

Probe Name: Has_circ_0008925–1:5′–AGAACTATTTTCCCTGTGATTTTA −3′; Has_circ_0008925–2:5′–ATTTTCCCTGTGATTTTATATCCA−3′; Has_circ_0008925–3: 5′–GCAAGCAGAACTATTTTCCTGTG−3′.

### 2.6 Animal Model and Adeno‐Associated Virus (AAV) Transfection

The animal experiments were approved by the Experimental Animal Welfare and Ethics Committee of Wannan Medical College (Approval number: LLSC‐2022‐142). C57 male mice (6–8 weeks, 25–30 g) were used to establish a UUO model according to previous studies [8]. Briefly, male C57BL/6J mice (weighing 20–25 g) were anaesthetised with intraperitoneal pentobarbital (35 mg/kg). The right ureter was ligated with 5–0 silk suture at two points after exposure. The mice were renal pelvic–injected with pAAV‐CAG‐mCherry‐3Xflag‐miR30shRNA (mmu_circ_0002215)‐WPRE (AAV‐ mmu_circ_0002215 shRNA) to inhibit the expression of mmu_circ_0002215 and either AAV‐negative control pAAV‐CAG‐mCherry‐3Xflag‐miR30shRNA (NC)‐WPRE (AAV‐NC) (5 × 10^10^ viral genome copy number, OBiO). After 7 days, the right ureter of the surviving mice was ligated to establish the UUO model. Indacaterol was injected intraperitoneally daily at a concentration of 2.5 mg/kg. After 7 days, the mice were euthanised, and the ligated kidneys were removed for subsequent experiments.

### 2.7 Immunofluorescence Staining

Paraffin sections (4 μm) of nephridial tissue or cell‐containing small dishes were fixed with 4% paraformaldehyde and permeabilised with 0.3%–0.5% Triton X‐100. Collagen I (diluted 1:100; Beyotime, China) and α‐SMA (diluted 1:100; Proteintech, USA) primary antibodies were used for staining. The secondary antibody used was Alexa Fluor 594‐conjugated anti‐rabbit IgG (Invitrogen, Carlsbad). Nuclei were detected using DAPI (Beyotime, China). Images were captured using a Leica TCS SP8 confocal laser scanning microscope.

### 2.8 Renal Histopathology

The kidney of each mouse was collected after sacrifice at week 1. Then, the kidneys were removed and prepared for tissue fixation by being wrapped in 4% paraformaldehyde overnight. Subsequently, the tissues were dehydrated with gradient alcohol and embedded in paraffin. Thin sections measuring 4 μm in thickness were cut from the nephridial tissues and stained with haematoxylin and eosin (H&E, Solarbio, China) to assess kidney morphological changes. Masson's trichrome (Solarbio, China) staining was utilised to quantify the extent of renal fibrosis.

### 2.9 Real‐Time Quantitative PCR (RT–qPCR)

According to the circBase database, the mouse homologous circRNA for human‐derived hsa_circ_0008925 is mmu_circ_0002215. Total RNA was extracted using TRIzol‐LS reagent (Thermo Fisher Scientific, USA) following the manufacturer's instructions. cDNA was synthesised using reverse transcription reagent (PrimeScript RT Reagent Kit, TaKaRa, Japan) and amplified by RT–qPCR (TB Green Premix Ex Taq, TaKaRa, Japan) to determine the expression of hsa_circ_0008925, with U6 serving as an internal reference. The primary primers used were as follows:

hsa_circ_0008925: forward primer: 5′–TTATGGCTGTCCTTGGGAGTTT‐3′, reverse primer: 5′–GGTATTCTCGGTCTGTTTTGGA–3′.

mmu_circ_0002215: forward primer: 5′–GGACATAAAATCACAGGGAAAAT–3′, reverse primer: 5′–ACTTCATCACCTCCTTTATCTGG–3′.

human‐SRSF6: forward primer: 5′–GCAAGCCTCCACTGCTTTTC–3′, reverse primer: 5′–CAAGGTAGACAAACCCGCCT–3′.

human‐U6: forward primer: 5′–GCTTCGGCAGCACATATACTAAAAT–3′, reverse primer: 5′–CGCTTCACGAATTTGCGTGTCAT–3′.

mouse‐U6: forward primer: 5’‐GCTTCGGCAGCACATATACTAAAAT–3′, reverse primer: 5′–CGCTTCACGAATTTGCGTGT CAT–3′.

### 2.10 Western Blot

The cells or tissues were lysed using a mixture of RIPA lysis buffer and phenyl methanesulphonyl fluoride at a ratio of 100:1. Next, a volume of loading buffer was added based on the sample density, and the samples were boiled in a metal bath for 10 min. Subsequently, the protein samples were subjected to electrophoresis and membrane transfer to a PVDF membrane. Primary antibodies, including rabbit collagen I (diluted 1:1000, Beyotime, China), α‐SMA (diluted 1:1500, Proteintech, USA) and SRSF6 (diluted 1:1500, Proteintech, USA), were added and incubated at 4°C overnight. After several washes, the membranes were incubated with a secondary antibody (goat anti‐rabbit IgG (H + L), diluted 1:40,000; Beyotime, China) for 2 h at room temperature. Following another wash, the developer was applied to the membrane, and detection was carried out using an automatic chemiluminescence image analysis system (Tanon 5200, China).

### 2.11 Statistical Analysis

The data were analysed using SPSS 26.0 software. RT–qPCR results were calculated using the 2^−ΔΔCt^ method. Normally distributed measurements are presented as x ± s, while non‐normally distributed measurements are presented as M (P25, P75). Group comparisons were conducted using t tests or rank‐sum tests; categorical count data are expressed as the number of cases, and group comparisons were made using *χ* ^2^ tests or Fisher's exact probability method. Spearman's correlation analysis was used for correlation analysis. A *p* value of < 0.05 was considered to indicate statistical significance.

## 3 Results

### 3.1 Magnetic Bead Sorting of Renal Tubular Epithelial Cells

**FIGURE 1:**
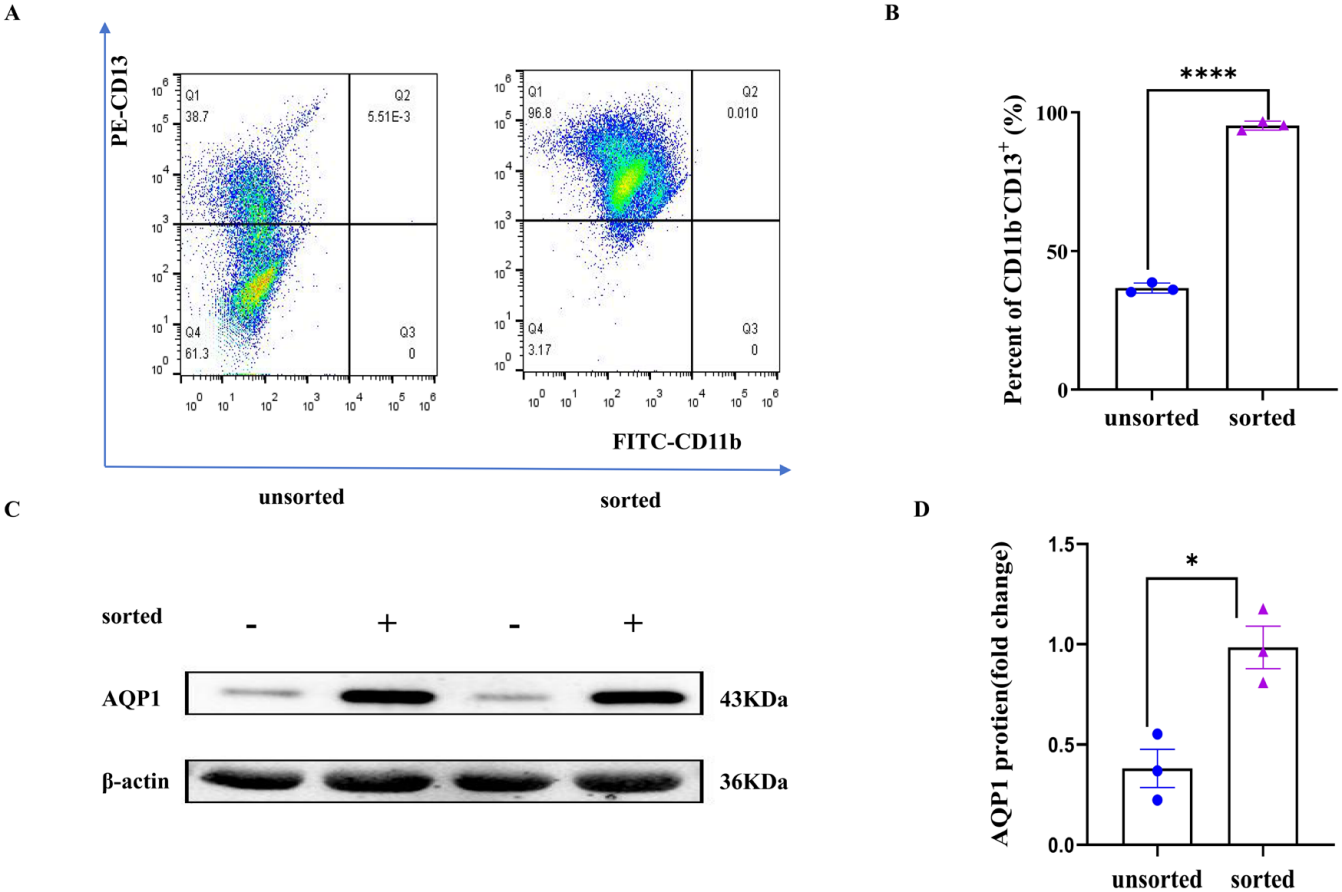
Magnetic bead sorting of renal tubular epithelial cells. (A) Representative flow cytometry images of sorted cells. (B) Mean percentage of CD11b^−^ CD13^+^ cells in sorted cells (*n* = 3). (C) Western blot results demonstrated the presence of AQP‐1 expression in the CD11b^−^ CD13^+^ cells. (D) Quantitative protein blotting plots of AQP‐1 (*n* = 3) (*p < 0.05; ****p < 0.0001).

Renal tubular epithelial cells were isolated from human urinary sediment using magnetic bead sorting, and the sorting outcomes were confirmed by flow cytometry and Western blot analysis. Flow cytometry indicated that the sorted cells exhibited CD13^+^ and CD11b^−^ phenotypes (Figure 1A). The percentage of CD13^+^ CD11b^−^ cells increased after sorting (36.7% ± 1.8% vs. 95.3% ± 1.6%, *p* < 0.0001; Figure 1B). AQP‐1 was utilised as a marker protein for identifying renal tubular epithelial cells. Western blot results demonstrated the presence of AQP‐1 in CD13^+^CD11b^−^ cells (Figure 1C,D).

### 3.2 Clinical and Pathological Data of Urinary Renal Tubular Epithelial Cells in Patients With CKD

**FIGURE 2:**
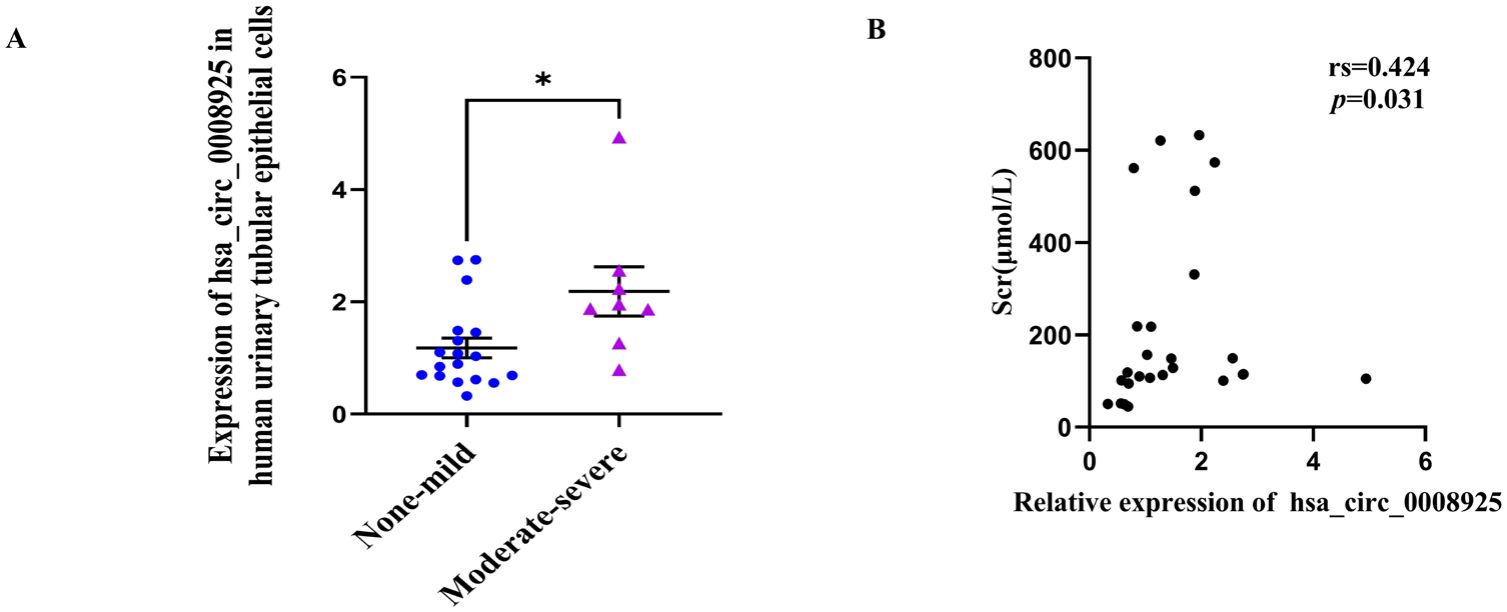
Clinical and pathological data of urinary renal tubular epithelial cells in patients with CKD. (A) RT‐qPCR analysis showed that the expression of human urinary renal tubular epithelial cell–derived hsa_circ_0008925 was significantly upregulated in the moderate–severe fibrosis group (*n* = 8) compared to that of the none–mild fibrosis group (*n* = 18). (B) Correlation analysis between the expression of has_circ_0008925 and Scr (*p < 0.05).

CKD patients were categorised into two groups based on the extent of renal fibrosis: the no fibrosis–mild fibrosis group (fibrosis area ≤ 25%, *n* = 18) and the moderate–severe fibrosis group (fibrosis area > 25%, *n* = 8). There were no statistically significant differences between the two groups in terms of age, sex, systolic blood pressure (SBP), or diastolic blood pressure (DBP) (*p* > 0.05). Scr, BUN, Cystatin C, and 24‐h proteinuria levels were greater in the moderate–severe fibrosis group than in the no fibrosis–mild fibrosis group, while the eGFR was lower in the moderate–severe fibrosis group (*p* < 0.001) (Table 1). The relative expression of tubular epithelial cell–derived hsa_circ_0008925 was significantly greater in the moderate–severe fibrosis group [median expression 1.923 (1.417–2.482)] than in the no fibrosis–mild fibrosis group [0.963 (0.664–1.460)] (*p* = 0.022) (Figure 2A). Correlation analysis indicated a positive correlation between the hsa_circ_0008925 expression level and the Scr level (rs = 0.424, *p* = 0.031); Figure 2B). For further details, please refer to Supplementary Clinical Material Table S1 and Figure S1.

**TABLE 1:** Clinical and pathological data of urinary renal tubular epithelial cells in patients with CKD.

	None–mild (*n* = 18)	Moderate–severe (*n* = 8)	*p*
Age (year)	46.3 ± 3.4	47.0 ± 5.2	0.916
Sex (male/female)	7/11	1/7	0.178
SBP (mmHg)	128.7 ± 5.1	135.0 ± 7.4	0.492
DBP (mmHg)	83.3 ± 3.4	84.6 ± 13.6	0.833
Scr (μmol/L)	113.3 ± 11.8	435.8 ± 75.2	0.001
BUN (mmol/L)	7.6 ± 0.6	15.3 ± 1.7	0.001
Cystatin C (mg/L)	1.4 ± 0.1	2.5 ± 0.3	0.001
eGFR (mL/min per 1.73 m^2^)	53.9 (41.8–80.9)	9.8 (8.8–37.4)	0.001
24 h proteinuria (g/day)	2.0 ± 0.3	4.3 ± 0.8	0.005
Hsa_circ_0008925	0.963 (0.664–1.46)	1.923 (1.417–2.482)	0.022
Score of TIF	—		0.001
Score of glomerular sclerosis	0.4 ± 0.1	0.5 ± 0.1	0.226

Abbreviations: BUN, blood urea nitrogen; eGFR, estimated glomerular filtration rate; Scr, serum creatinine.

### 3.3 TGF‐β1 Induces Increased hsa_circ_0008925 Expression in HK‐2 Cells

**FIGURE 3:**
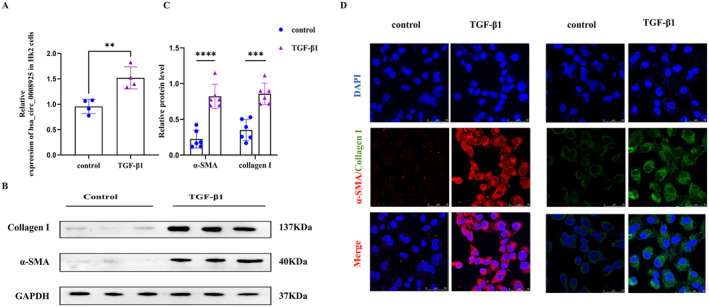
TGF‐β1 induces hsa_circ_0008925 expression level, which is elevated in HK‐2 cells. (A) RT‐qPCR analysis shows relative levels of hsa_circ_0008925 expression in HK2 cells (*n* = 4). (B) Western blot analysis of collagen I and α‐SMA protein levels in HK2 cells. (C) Quantitative protein blotting plots of collagen I and α‐SMA (*n* = 6). (D) Immunofluorescence images show α‐SMA and collagen I in HK2 cells (bar = 50 μm) (**p < 0.01; ***p < 0.001; ****p < 0.000).

After treatment with 15 ng/mL of TGF‐β1 for 48 h, circRNAs and protein were extracted for further analysis. RT–qPCR revealed an increase in the expression level of hsa_circ_0008925 in the TGF‐β1‐treated cells (Figure 3A). Additionally, Western blot analysis revealed elevated levels of the fibrosis‐related proteins collagen I and α‐SMA following TGF‐β1 administration (Figure 3B,C). Consistent with these findings, the immunofluorescence results demonstrated an increased expression of collagen I and α‐SMA in TGF‐β1‐treated HK2 cells (Figure 3D).

### 3.4 Hsa_circ_0008925 Silencing Attenuates TGF‐β1‐Induced Expression of Collagen I and α‐SMA in HK‐2 Cells

**FIGURE 4:**
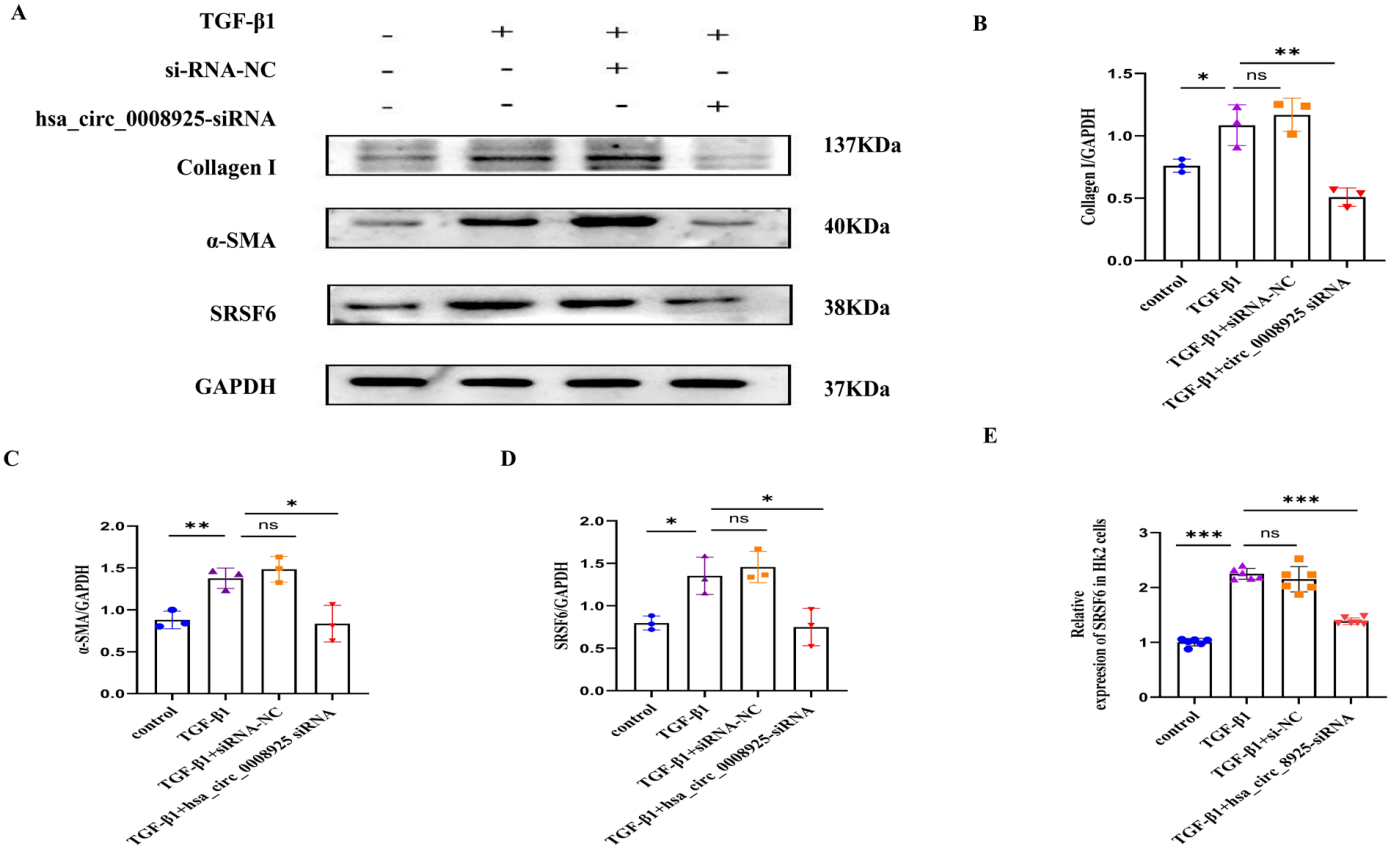
Hsa_circ_0008925 silencing attenuates TGF‐β1‐induced expression of collagen I, α‐SMA and SRSF6 in HK‐2 cells. (A) Western blot showed that protein expression of collagen I, α‐SMA and SRSF6 was inhibited by silencing hsa_circ_0008925 after transfection of siRNA in TGF‐β1‐treated HK2 cells. (B) Quantitative protein blotting plots of collagen I (*n* = 3). (C) Quantitative protein blotting plots of α‐SMA (*n* = 3). (D) Quantitative protein blotting plots of SRSF6 (*n* = 3). (E) RT‐qPCR analysis of the relative expression of SRSF6 mRNA in HK2 cells silenced hsa_circ_0008925 (*n* = 6) (ns > 0.05; *p < 0.05; **p < 0.01; ***p < 0.001).

**FIGURE 5:**
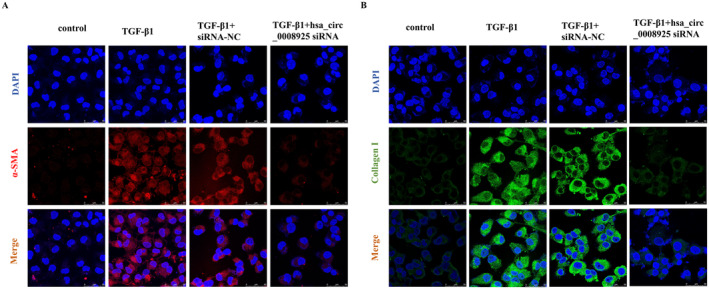
Hsa_circ_0008925 silencing attenuates TGF‐β1‐induced expression of collagen I, α‐SMA in HK‐2 cells. (A) A decreased α‐SMA fluorescence was detected by immunofluorescence confocal microscopy in HK2 cells silenced hsa_circ_0008925 after transfection with siRNA (bar = 50 μm). (B) Decreased collagen I fluorescence was detected by immunofluorescence confocal microscopy in HK2 cells silenced hsa_circ_0008925 after transfection with siRNA (bar = 50 μm).

To investigate the impact of hsa_circ_0008925 silencing on fibrogenesis, we transfected HK‐2 cells with hsa_circ_0008925 siRNA. The efficacy of hsa_circ_0008925 silencing was confirmed by RT–qPCR. Three siRNA sequences targeting different sites of hsa_circ_0008925 were tested. The most effective sequence was selected (Figure S2). Western blot (Figure 4A–C) and immunofluorescence (Figure 5) results showed a significant reduction in the fibrosis‐related marker proteins α‐SMA and collagen I after silencing hsa_circ_0008925 compared to those in the TGF‐β1 group.

### 3.5 RNA Pull‐Down Analysis

We conducted RNA pull‐down experiments to identify proteins that interact with hsa_circ_0008925. Mass spectrometry (MS) analysis indicated that SRSF6 may be a target protein of hsa_circ_0008925, a finding that we further validated through Western blotting. Both results demonstrated a significant elevation in the SRSF6 protein expression in TGF‐β1‐treated HK2 cells (Figure S3). The data obtained from MS analyses are detailed in Table S2.

### 3.6 Hsa_circ_0008925 Silencing Inhibits SRSF6 Expression

After HK‐2 cells were treated with hsa_circ_0008925 siRNA, RT–qPCR (Figure 4E) and Western blotting (Figure 4A) revealed decreases in the SRSF6 mRNA and protein levels.

### 3.7 The Expression of Renal Fibrosis Protein Markers After hsa_circ_0008925 Silencing and SRSF6 Inhibition

**FIGURE 6:**
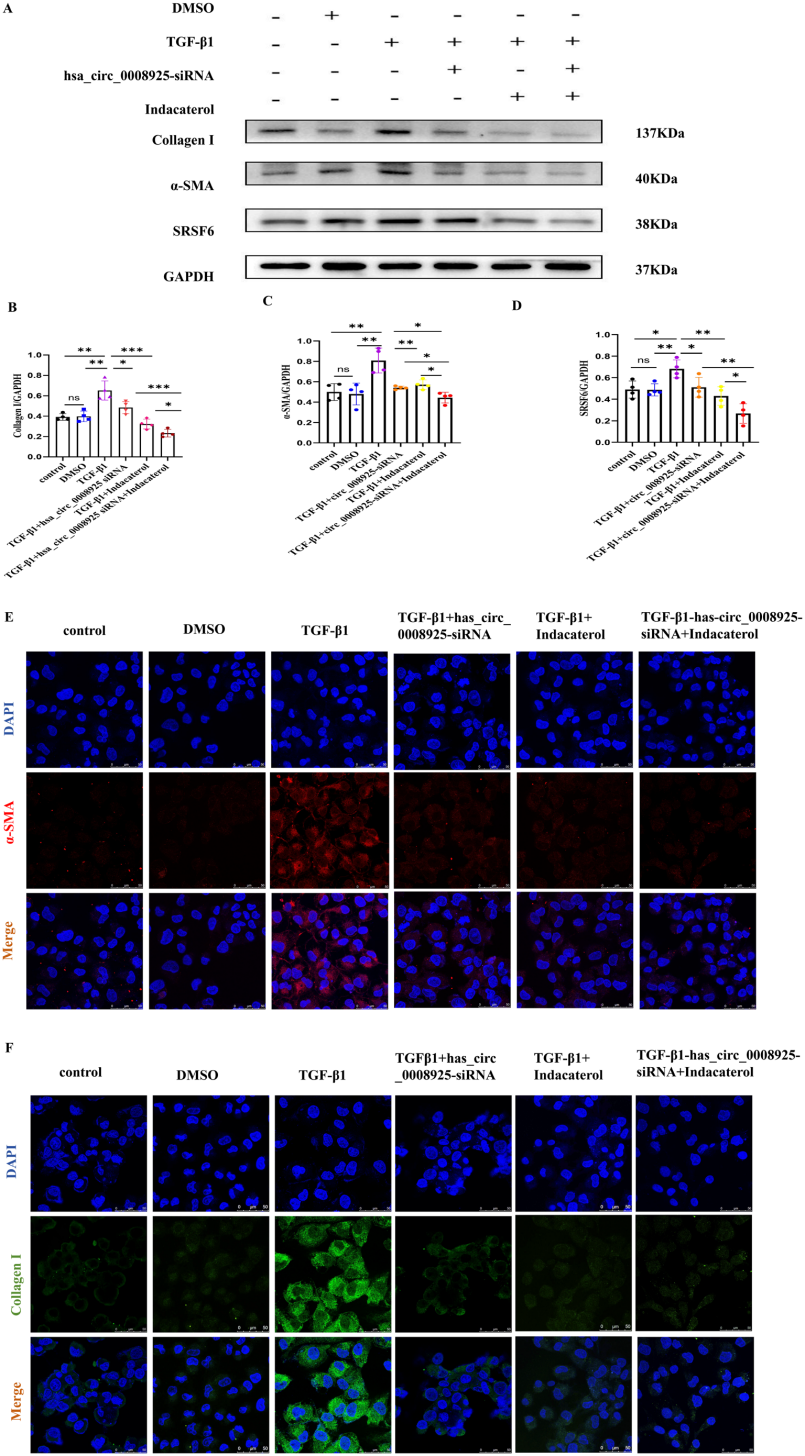
The expression of renal fibrosis protein markers after hsa_circ_0008925 silencing and SRSF6 inhibiting. (A) Western blot demonstrated the inhibition of protein expression of collagen I, α‐SMA and SRSF6 by silencing hsa_circ_0008925 after transfection of siRNA and addition of indacaterol in TGF‐β1‐treated HK2 cells. (B) Quantitative protein blotting plots of collagen I (*n* = 4). (C) Quantitative protein blotting plots of α‐SMA (*n* = 4). (D) Quantitative protein blotting plots of SRSF6 (*n* = 4). (E) Immunofluorescence images show α‐SMA after transfection of siRNA and addition of indacaterol in TGF‐β1‐treated HK2 cells (bar = 50 μm). (F) Immunofluorescence images show collagen I after transfection of siRNA and addition of indacaterol in TGF‐β1‐treated HK2 cells (bar = 50 μm) (*p < 0.05; **p < 0.01; ***p < 0.001).

Treatment with hsa_circ_0008925 siRNA significantly decreased the expression of collagen I and α‐SMA. Administration of indacaterol did not only inhibit the expression profile of SRSF6 protein but also inhibit collagen I and α‐SMA expression (Figure 6A–D). Immunofluorescence showed that after treatment with hsa_circ_0008925 siRNA and indacaterol, the expression of α‐SMA (Figure 6E) and collagen I (Figure 6F) significantly decreased in HK‐2 cells. Results were consistent with the knockdown of hsa_circ_0008925 expression.

### 3.8 Silencing mmu_circ_0002215 and Inhibiting SRSF6 Alleviate Renal Fibrosis in UUO Model

**FIGURE 7:**
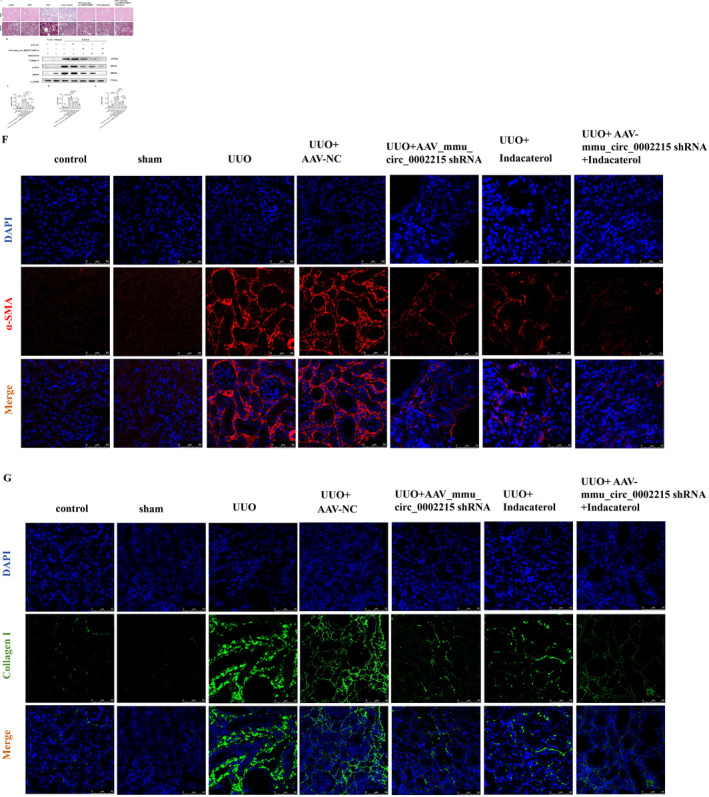
Silencing of mmu_circ_0002215 and inhibiting of SRSF6 alleviated renal fibrosis in UUO model. (A) Haematoxylin and eosin (H&E) and Masson trichrome staining images of the mouse kidney (bar = 20 μm). (B) Inhibition of protein expression of collagen I, α‐SMA and SRSF6 by silencing mmu_circ_0002215 after transfection of shRNA and addition of indacaterol in mouse kidney. (C) Quantitative protein blotting plots of collagen I (*n* = 4). (D) Quantitative protein blotting plots of α‐SMA (*n* = 4). (E) Quantitative protein blotting plots of SRSF6 (*n* = 4). (F) Representative images of immunofluorescence of α‐SMA proteins in mouse kidney (bar = 50 μm). (G) Representative images of immunofluorescence of collagen I proteins in mouse kidney (bar = 50 μm) (*p < 0.05;**p < 0.01; ***p < 0.001; ****p < 0.0001).

To further validate the involvement of hsa_circ_0008925 in the regulation of renal fibrosis through the SRSF6 pathway, we conducted in vivo experiments using a UUO mouse model. According to the circBase database, the mouse homologous circRNA of hsa_circ_0008925 is mmu_circ_0002215. Three AAV‐targeting different sites of mmu_circ_0002215 were constructed. Immunofluorescence showed stable red fluorescence in the renal tissue after AAV injection. The most effective AAV silencing agent was selected (Figure S4). After the transfection of AAV‐mmu_circ_0002215, a UUO model was established, and an intraperitoneal injection of indacaterol at a dosage of 2.5 mg/kg per day was administered. After 7 days, the mice were euthanised, and the kidneys were collected for analysis. HE staining revealed that compared with the control group, the UUO and AAV‐NC groups exhibited significant inflammatory cell infiltration in the inter‐renal compartment, renal tubular epithelial cell atrophy and tubular lumen dilatation. Conversely, inflammatory renal injury was mitigated by either AAV‐mmu_circ_0002215 transfection and indacaterol supplementation. Compared with those in the UUO group, Masson trichrome staining revealed minimal accumulation of the blue collagen tissue in the renal tissue surrounding the perivascular area in the UUO + AAV‐mmu_circ_0002215 group, UUO + indacaterol group and UUO + AAV‐mmu_circ_0002215 + indacaterol group (Figure 7A).

### 3.9 Silencing mmu_circ_0002215 and Inhibiting SRSF6 Decrease the Expression of Renal Fibrosis Marker Proteins

Western blot analysis and tissue immunofluorescence staining showed that AAV‐mmu_circ_0002215 transfection significantly decreased the expression of collagen I, α‐SMA and SRSF6. Furthermore, the administration of indacaterol reversed the progression of renal fibrosis, effectively reducing the development of renal interstitial fibrosis (Figure 7B–E). Immunofluorescence showed that the expression of collagen I and α‐SMA decreased in the kidney tissue after AAV‐mmu_circ_0002215 transfection and the administration of indacaterol (Figure 7F,G).

## 4 Discussion

This study focused on investigating the profibrotic effects of hsa_circ_0008925 and examining the potential mechanisms involved in regulating renal fibrosis through cell and animal experiments. We found that the expression of hsa_circ_0008925 was increased in urinary tubular epithelial cell–derived renal cells from patients with renal fibrosis. Hsa_circ_0008925 promoted renal fibrosis by upregulating the expression of SRSF6 in vitro and in vivo. These findings indicate that targeting hsa_circ_0008925/SRSF6 could be a promising therapeutic approach for managing renal fibrosis.

Renal fibrosis is a critical pathological process in the progression of CKD to ESRD. Early diagnosis is crucial for slowing the progression of CKD [9]. As a noninvasive source reflecting pathological changes in the kidney, urine can be used as a biomarker for diagnosing and predicting renal disease [10, 11]. The biological information contained in urine can also reflect the pathological and physiological conditions of kidney diseases. Baer et al. [12] successfully purified CD13^−^‐labelled renal tubular epithelial cells from the renal tissue using immunomagnetic bead technology. Previous studies have also revealed biological information related to renal epithelial–mesenchymal transition (EMT) in renal tubular epithelial cells in the urinary sediment of CKD patients [13, 14, 15]. In this study, urine‐derived renal tubular epithelial cells were sorted by immunomagnetic beads, and we further found that the hsa_circ_0008925 expression was upregulated in renal fibrosis patients. These data indicated that the renal tubular epithelial cell hsa_circ_0008925 may participate in the progression of renal fibrosis.

CircRNAs are a distinct class of RNA molecules known for their stability and tissue‐specific expression patterns, attributed to their characteristic covalently closed loop structure. In the field of renal fibrosis, circRNAs have demonstrated tremendous research potential due to their multifunctionality in disease progression [16, 17, 18]. A previous study of a UUO‐induced renal fibrosis model revealed that circACTR2 activates the NLRP3 inflammasome, leading to macrophage apoptosis and the release of inflammatory factors by sponging miR‐561 [19]. Additionally, research has shown that an increasing number of circRNAs are associated with kidney disease [20]. The chromosomal location of hsa_circ_0008925 is chr6:108222573–108246136, and its gene name is SEC63 [21]. No studies have reported the relationship between hsa_circ_0008925 and renal fibrosis. We identified a new circRNA in renal tubular cells from renal fibrosis patients that regulates renal fibrosis by directly separating renal tubular cells from urine. In vitro experiments using the HK‐2 cell line also confirmed that hsa_circ_0008925 regulates renal fibrosis.

CircRNAs not only function through competing endogenous RNAs (ceRNAs) but also directly regulate the protein expression and exert their effects on organ disorders [22]. Our findings suggest that hsa_circ_0008925 potentially contributes to the development of renal fibrosis by interacting with SRSF6 proteins. SRSF6 is a member of the serine/arginine‐rich (SR) family of proteins. SRSF6 plays an important role in various stages of tumour development and progression by controlling the splicing of oncogenes and tumour suppressor genes [23]. SRSF6 also participates in other biological processes, such as transcriptional regulation and protein stability [24]. Liang et al. [25] reported that treating pleural mesothelial cells (PMCs) with bleomycin and TGF‐β1 increased SRSF6 levels. Elevated SRSF6 promoted PMC proliferation and increased the synthesis of the fibrotic proteins COL1A2 and ACTA2. Manetti et al. [26] revealed that SRSF6 is involved in the fibrotic process in systemic sclerosis patients by regulating the expression of vascular endothelial growth factor (VEGF). Although the role of SRSF6 in various fibrotic diseases has gradually emerged, its specific involvement in renal fibrosis remains unclear. In our study, we discovered that SRSF6 has a profibrotic role in renal fibrosis, indicating its involvement not only in tumours but also in the progression of renal fibrosis. These findings offer new insights into the molecular mechanisms underlying renal fibrosis and could serve as a foundation for developing therapeutic approaches targeting SRSF6 for treating renal fibrosis. Indacaterol is a β2 agonist originally developed for the treatment of chronic obstructive pulmonary disease (COPD). While investigating the molecular function of SRSF6 in colorectal cancer (CRC) progression, Wang et al. [27] discovered that indacaterol competitively inhibits RNA binding to SRSF6, exhibiting an antagonistic effect on SRSF6 splicing regulation. Following treatment with indacaterol, we observed not only a suppression of SRSF6 protein expression but also a significant inhibition of renal fibrogenesis. These results are consistent with the knockdown of the hsa_circ_0008925 gene. This further validates the involvement of the hsa_circ_0008925/SRSF6 signalling pathway in renal fibrosis and suggests that indacaterol may serve as a novel strategy for treating renal fibrosis through the targeting of SRSF6.

Our study has several limitations. First, due to the lack of follow‐up studies, the impact of hsa_circ_0008925 on patient prognosis and its predictive value for the prognosis of renal fibrosis are unclear. Furthermore, the lack of identification of a specific hsa_circ_0008925 fragment that binds to SRSF6 prevents a comprehensive exploration of the molecular mechanism involving hsa_circ_0008925 and SRSF6. Finally, this study focused solely on the hsa_circ_0008925/SRSF6 pathway without delving into the splicing target genes downstream of this pathway.

## Author Contributions

**Yuanhui Shi:** data curation (lead), formal analysis (lead), writing – original draft (supporting). **Yuye Chen:** investigation (equal), software (equal). **Zihao Xiao:** investigation (supporting), resources (lead). **Yajie Wang:** formal analysis (supporting), software (supporting). **Cong Fu:** conceptualization (supporting), funding acquisition (supporting), writing – review and editing (equal). **Yuhan Cao:** conceptualization (supporting), funding acquisition (supporting), writing – review and editing (lead).

## Conflicts of Interest

The authors declare no conflicts of interest.

## Supporting information


**Figure S1.** Correlation between the hsa_circ_0008925 expression in renal tubular epithelial cells and renal function. (A) Correlation between hsa_circ_0008925 and BUN (rs = 0.151，*p* = 0.463). (B) Correlation between hsa_circ_0008925 and Cystatin C (rs = 0.201，*p* = 0.325). (C) Correlation between hsa_circ_0008925 and eGFR (rs = 0.293，*p* = 0.147). (D) Correlation between hsa_circ_0008925 and 24 h proteinuria (rs = 0.0119，*p* = 0.563).
**Figure S2**. RT‐qPCR analysis shows that siRNA transfected with HK2 cells knocked down the relative expression level of hsa_circ_0008925 (*n* = 3, ns > 0.05;**p < 0.01; ***p < 0.001).
**Figure S3**. (A) Western blot and quantification of SRS6 in TGF‐β1‐treated HK2 cells. (B) Quantitative protein blotting plots of SRSF6 (*n* = 6, ***p < 0.001).
**Figure S4**. (A) Confocal immunofluorescence demonstrates that AAV can be successfully transfected into mouse kidneys by renal pelvic injection. (B) RT‐qPCR showed that all inhibited the expression of mmu_circ_0002215 in mouse kidney, and the best transfection efficiency was achieved with the AAV1‐mmu_circ_0002215 shRNA‐1 W knockdown sequence (*n* = 3, *p < 0.05).

**Table S1.** Clinical and pathological data of all selected recipients.

**Table S2.** The data obtained from mass spectrometry analyses are detailed in Table S2. This section presents the differential protein expression analyses conducted following the TGF‐β1 treatment, primarily comparing the treated samples to the control samples. Generally, the protein species that exhibit higher abundance in the treated samples compared to those in the control samples are highlighted.

## Data Availability

The data that support the findings of this study are available in the methods and/or Supporting Information of this article.
