# Supplementary figures and images for: Renal Tubular Epithelial Cell–Derived hsa_circ_0008925 From Urine Is Related to Chronic Renal Fibrosis
Source: J Cell Mol Med. 2025 Jan 12;29(1):e70335. doi: 10.1111/jcmm.70335 (PMC11725181; doi:10.1111/jcmm.70335)

Figure S1

A

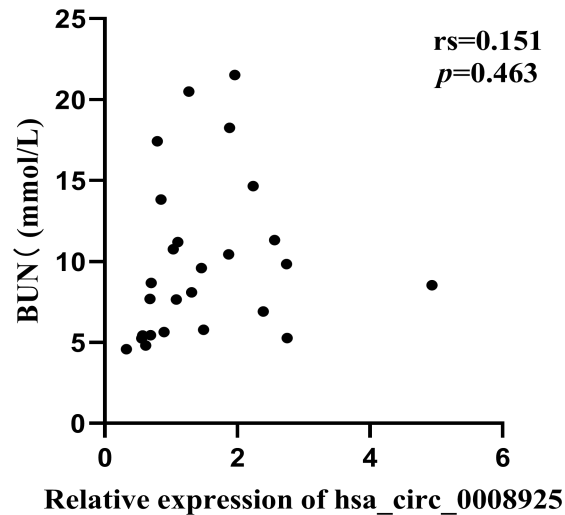

B

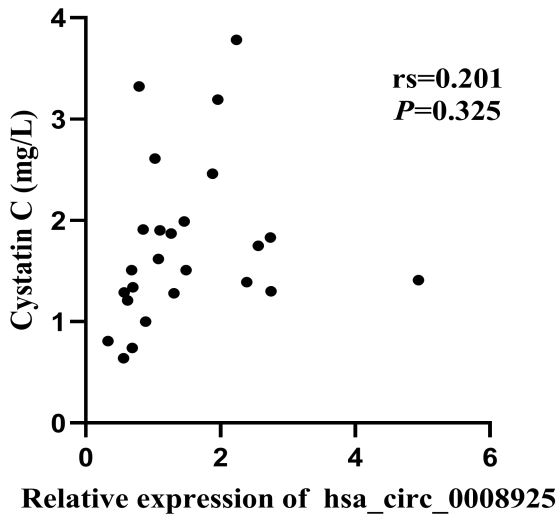

C

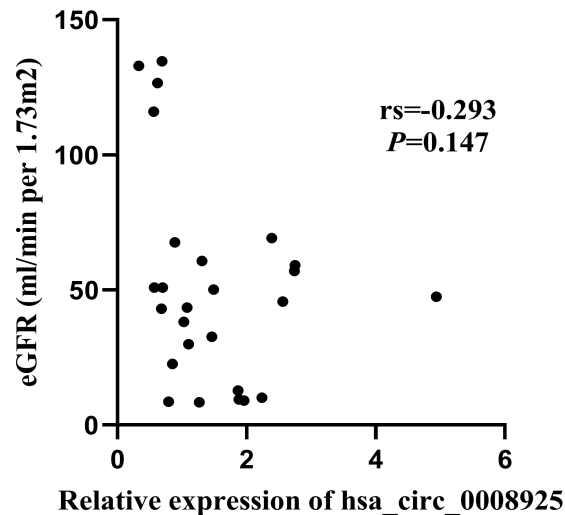

D

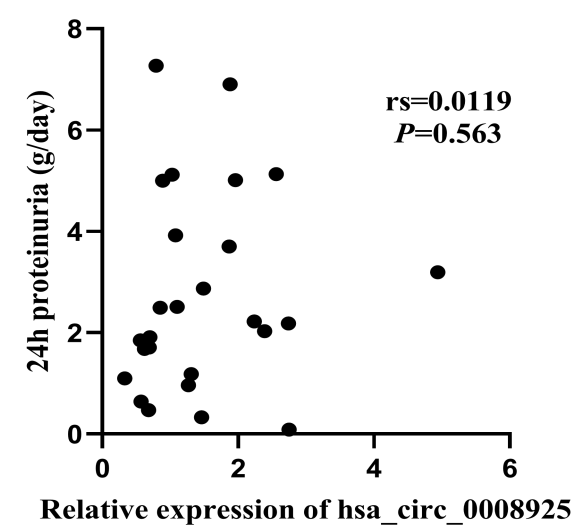

Figure S2

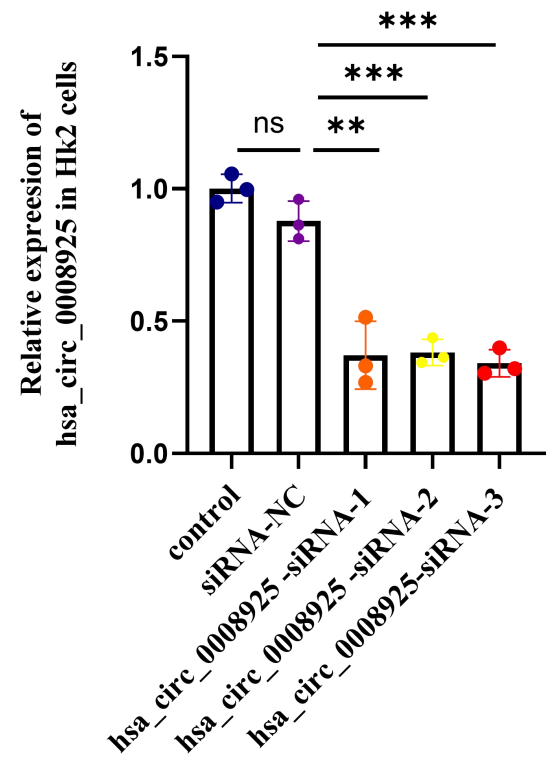

Figure S3

A

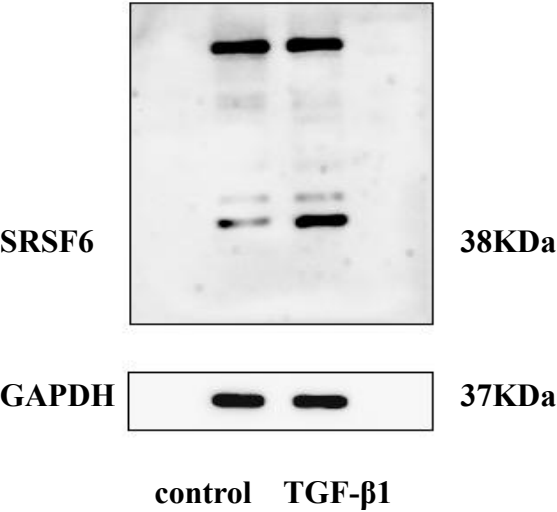

B

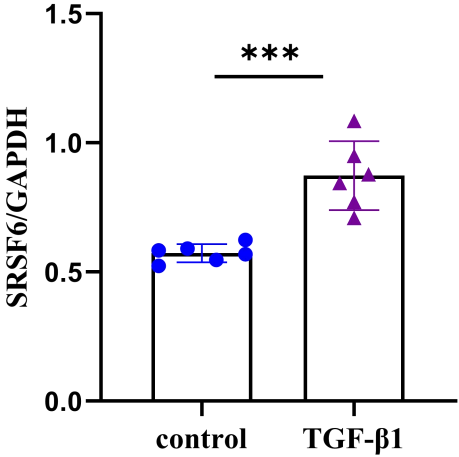

Figure S4

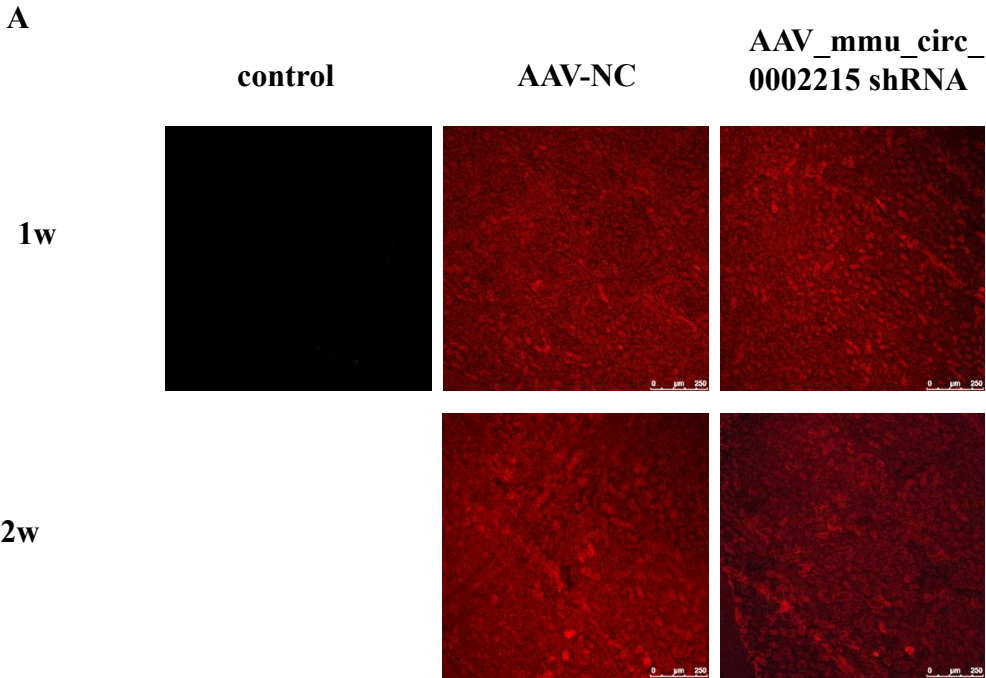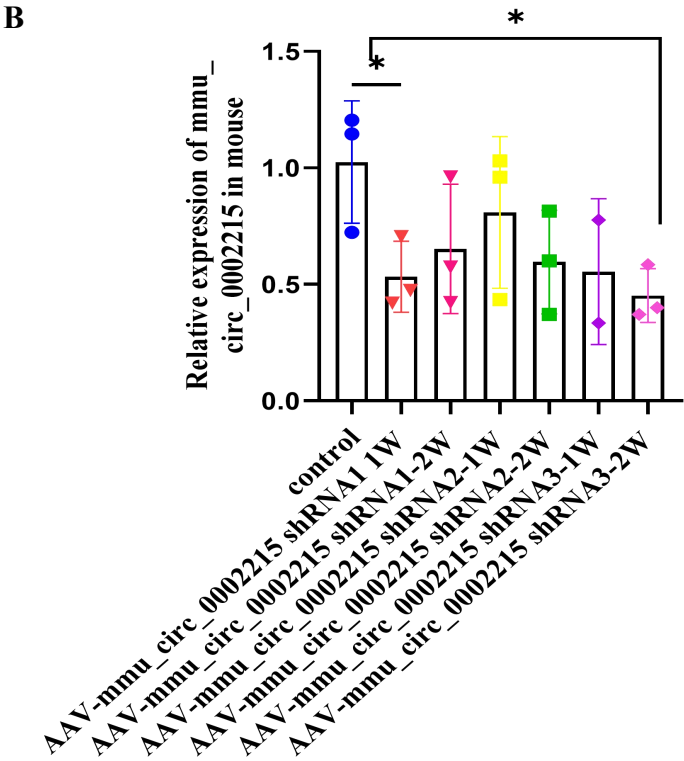

Supplement: Supplementary file 1 — Figure S1. Correlation between the hsa_circ_0008925 expression in renal tubular epithelial cells and renal function. (A) Correlation between hsa_circ_0008925 and BUN (rs = 0.151，p = 0.463). (B) Correlation between hsa_circ_0008925 and Cystatin C (rs = 0.201，p = 0.325). (C) Correlation between hsa_circ_0008925 and eGFR (rs = 0.293，p = 0.147). (D) Correlation between hsa_circ_0008925 and 24 h proteinuria (rs = 0.0119，p = 0.563). Figure S2. RT‐qPCR analysis shows that siRNA transfected with HK2 cells knocked down the relative expression level of hsa_circ_0008925 (n = 3, ns > 0.05;**p < 0.01; ***p < 0.001). Figure S3. (A) Western blot and quantification of SRS6 in TGF‐β1‐treated HK2 cells. (B) Quantitative protein blotting plots of SRSF6 (n = 6, ***p < 0.001). Figure S4. (A) Confocal immunofluorescence demonstrates that AAV can be successfully transfected into mouse kidneys by renal pelvic injection. (B) RT‐qPCR showed that all inhibited the expression of mmu_circ_0002215 in mouse kidney, and the best transfection efficiency was achieved with the AAV1‐mmu_circ_0002215 shRNA‐1 W knockdown sequence (n = 3, *p < 0.05). [file JCMM-29-e70335-s001.pdf]
